# Expression of Dopamine D_1−4_ and Serotonin 5-HT_1A-3A_ Receptors in Blood Mononuclear Cells in Schizophrenia

**DOI:** 10.3389/fpsyt.2021.645081

**Published:** 2021-03-10

**Authors:** Adam Wysokiński, Elżbieta Kozłowska, Ewa Szczepocka, Anna Łucka, Justyna Agier, Ewa Brzezińska-Błaszczyk, Katarzyna Sobierajska

**Affiliations:** ^1^Department of Old Age Psychiatry and Psychotic Disorders, Medical University of Lodz, Lodz, Poland; ^2^Department of Experimental Immunology, Medical University of Lodz, Lodz, Poland; ^3^Department of Molecular Cell Mechanisms, Medical University of Lodz, Lodz, Poland

**Keywords:** Schizophrenia, dopamine receptors, serotonin receptors, PBMCs, biomarkers

## Abstract

**Introduction:** The aim of this study was to determine the mRNA expression profile of dopamine D_1_, D_2_, D_3_, D_4_ and serotonin 5-HT_1A_, 5-HT_2A_, and 5-HT_3A_ receptors in peripheral blood mononuclear cells (PBMCs) in schizophrenia and the *in vitro* effect of antipsychotics on the expression of these receptors in PBMCs of healthy subjects.

**Materials and Methods:** Twenty-seven patients with schizophrenia and 29 healthy controls were recruited for the study. All study subjects underwent thorough clinical assessment, including anthropometric and body composition measurements. The expression of mRNA for dopamine D_1−4_ and serotonin 5-HT_1A−3A_ receptors was measured using quantitative RT-PCR in peripheral blood mononuclear cells. *In vitro* mRNA and protein expression of these receptors was measured using quantitative RT-PCR and Western Blotting in PBMCs cultured with quetiapine, haloperidol, aripiprazole, risperidone, olanzapine or clozapine at IC_50_, half of IC_50_, and one-quarter of IC_50_ concentrations.

**Results:** The key finding was that the schizophrenia group demonstrated significantly higher mRNA expression of D_1_, D_2_ and D_4_ receptors (*p* < 0.001), and significantly lower mRNA expression of 5-HT_3A_ receptors (*p* < 0.01). After adjusting for smoking, the mRNA expression of D_1_ lost its significance, while that of D_3_, 5-HT_1A_, 5-HT_2A_ became significant (all three were lower in the schizophrenia group). These receptors also demonstrated different ratios of mRNA expression in the schizophrenia group. The *in vitro* experiments showed that high concentrations of antipsychotics influenced the mRNA and protein expression of all studied receptors.

**Conclusion:** Schizophrenia patients display a distinctive pattern of dopamine and serotonin receptor mRNA expression in blood mononuclear cells. This expression is little affected by antipsychotic treatment and it may therefore serve as a useful diagnostic biomarker for schizophrenia.

## Introduction

Schizophrenia is a chronic and severe neurodevelopmental mental disorder characterized by impairments in multiple domains of everyday life, which results in a higher chance of disability (1), excessive early mortality (2), and a high prevalence of treatment resistance (3). While great efforts have been made to identify clinically applicable neuronal biomarkers of schizophrenia, no such diagnostic indicators or predictors of treatment response have been identified so far (4). Therefore, interest has grown in the use of peripheral, usually blood-based, biomarkers as valid alternatives. Peripheral blood mononuclear cells (PBMCs), which consist of lymphocytes (T cells, B cells, NK cells) and monocytes, serve as a potential source for peripheral biomarkers.

Numerous studies have found that dopamine and serotonin neurotransmitter systems play a significant role in the pathophysiology of schizophrenia. These two systems are involved in all major domains of schizophrenia symptoms (positive, negative, cognitive, and affective). Also, all antipsychotics exert complex effects upon a range of neurotransmitters including their receptors. There are at least five subtypes of dopamine receptors: D_1_, D_2_, D_3_, D_4_, and D_5_. Of these, D_1_ and D_5_ are members of the D_1_-like family, while D_2_, D_3_, and D_4_ belong to the D_2_-like family (5). D_1_-like receptors activate adenylyl cyclase, increasing the intracellular concentration of the second messenger cyclic adenosine monophosphate (cAMP), while D_2_-like receptors inhibit the production of cAMP. Therefore, D_1_-like receptors are classified as excitatory dopamine receptors, and D_2_-like receptors as inhibitory dopamine receptors. At least seven families of serotonin receptors have been identified (5-HT_1−7_), with several subtypes in each family.

The negative and cognitive symptoms of schizophrenia may be elicited by reduced stimulation of D_1_ receptors (6). However, patients with schizophrenia have been found to demonstrate increased D_1_ receptor availability, and this might constitute a compensatory, but ineffective, up-regulation mechanism, secondary to sustained deficiency in mesocortical dopamine function (7). The dopamine hypothesis of schizophrenia attributes positive (psychotic) symptoms to increased density and activity of D_2_ and D_3_ receptors, as well as increased presynaptic dopamine availability (8). Also, a recent study by Veselinovic et al. found that dopamine D_2_ and D_3_ receptors may influence cognitive functions in schizophrenia (9). Despite having the lowest level of expression in the brain, D_4_ receptors are of particular interest, since they have a high affinity to the atypical neuroleptic clozapine. It has been proposed that clozapine treatment may act on schizophrenia through D4 receptor-mediated GABA modulation, suggesting that excitatory transmission is disinhibited in the intrinsic cortical, thalamocortical and extrapyramidal pathways (10). Also, D_4_ receptors may be involved in the homeostatic regulation of glutamatergic transmission, which is dysregulated in acute psychosis (11).

There are two well-replicated postmortem findings concerning the serotonergic system in schizophrenia: reduced serotonin 5-HT_2A_ receptor expression within the frontal cortex and increased density of 5-HT_1A_ receptors (12). These alterations may underlie psychotic symptoms and cognitive dysfunctions, and may determine the response to antipsychotic treatment (13). 5-HT_2A_ receptors are acknowledged as being involved in the mechanism of action of second-generation antipsychotics (which are antagonists of these receptors). However, selective 5-HT_2A_ antagonists are only marginally superior to placebo and inferior to conventional antipsychotics (14). The role of 5-HT_3A_ receptors is less understood. Selective 5-HT_3_ antagonists (e.g., ondansetron and tropisetron) have been shown to improve P_50_ auditory gating in schizophrenia and may be used to enhance neurocognitive deficits (15).

While the expression of D_1−5_ (16–18) and 5-HT_1A_ (19), 5-HT_2A_ (20), 5-HT3A (21) receptors has previously been confirmed in PBMCs, few studies have measured the expression of these receptors in PBMCs of schizophrenia patients. There is an increasing body of evidence suggesting that blood lymphocytes may act as convenient indictors for certain cellular functions, including the expression of dopamine and serotonin receptor genes (22). Moreover, it has also been shown that peripheral blood lymphocytes possess dopamine (23) and serotonin (24) plasma membrane transporters, which may indicate pathologies characterized by impaired dopaminergic or serotonergic neurotransmission. Peripheral gene expression has been found to be a useful potential surrogate for gene expression in the brain (25). Therefore, the main aim of this study was to determine the mRNA expression profile of dopamine D_1_, D_2_, D_3_, and D_4_, and serotonin 5-HT_1A_, 5-HT_2A_, and 5-HT_3A_ receptors in PBMCs in patients with chronic schizophrenia. In addition, a secondary objective was to evaluate the relationships between these mRNA profiles and the clinical features of schizophrenia. As obesity is associated with peripheral inflammation and the down-regulation of dopaminergic pathways in PBMCs (26), the analysis also included detailed covariates of metabolic status established using anthropometric and body composition analysis. We propose that the expression of these receptors in PBMCs could be used as potential peripheral biomarkers of schizophrenia. As treatment with antipsychotics may affect the peripheral expression of selected receptors, the study includes additional *in vitro* experiments to assess mRNA and protein expression in the PBMCs of non-schizophrenic subjects cultured with six commonly-used antipsychotics. The study also evaluates the cytotoxic activity of these antipsychotics on PBMCs based on their ability to inhibit PBMC growth (half-maximal inhibitory concentrations; IC_50_).

## Materials and Methods

### Subjects

Twenty-seven Caucasian patients (age 18–60 years) with paranoid schizophrenia were enrolled into this cross-sectional study. Recruitment was performed in our hospital outpatient department: invitations were distributed to patients via the department doctors. All study patients were required to have an established diagnosis of paranoid schizophrenia. Recruitment was consecutive, with no randomization. Prior to enrollment, they underwent a semi-structured interview based on the ICD-10 criteria of schizophrenia to confirm the diagnosis. Twenty-nine healthy volunteers (recruited among the hospital personnel) were also randomly selected as a control group. All interviews were performed by the same doctor; therefore, an inter-rater reliability test was not carried out. All healthy volunteers were interviewed with the use of an ICD-10-based, semi-structured medical interview. For healthy controls, the exclusions criteria included: self-reported personal or familial history of mental disorders and any previous psychiatric treatment. Normal physical, neurological and endocrinological status was evaluated based on physical examination and laboratory tests (complete blood count, C-reactive protein, renal and hepatic markers and electrolyte levels). For all study subjects, the exclusion criteria included: immunological disorders (e.g., allergy, asthma, or AIDS), acute or chronic inflammatory conditions (e.g., pneumonia or rheumatoid arthritis), systemic diseases, or cancer. Also, all subjects with active alcohol or drug abuse (based on the interview) were excluded from the study.

All study subjects had been informed about the study procedures and expressed their written informed consent. As there were no grossly psychotic, disorganized or catatonic patients, all patients included into the study were capable of giving their informed consent. The study protocol was approved by the Bioethics Commission of the Medical University of Lodz (RNN/122/16/KE and RNN/370/19/KE).

### Clinical Assessments

All study patients had been interviewed using the Positive and Negative Syndrome Scale (PANSS) and Calgary Depression Scale for Schizophrenia (CDSS) scales. A single trained rater performed all assessments for all scales (E.Sz or A.Ł). The severity of schizophrenia symptoms was evaluated based on PANSS positive (P), negative (N), and general (G) sub-scales. According to PANSS scores patients were stratified as having dominating psychotic (POS+ group: PANSS P score > PANSS N score) or negative symptoms (NEG+ group: PANSS P score < PANSS N score). CDSS was used to measure the depressive symptoms and to classify patients as depressed (DEP+ group: CDSS score >6) or non-depressed (DEP- group) (27). We did not evaluate the inter-rater reliability for assessments.

### Laboratory Measurements

Blood samples were collected between 8 a.m., and 9 a.m., after at least an 8-h overnight fast. Serum glucose and lipid levels were measured using a Dirui CS-400 analyzer (Dirui, China). CRP serum levels were determined in fresh blood using latex enhanced immunoturbidimetric assay.

#### Isolation of PBMCs

PBMCs were obtained from whole blood collected from patients with schizophrenia and healthy subjects. For sulforhodamine assay and cell culture with drugs, PBMCs obtained from buffy coat collected from healthy donors, received as waste material from the Regional Center for Blood Donation and Blood Treatment (Łódz) were used. The density gradient centrifugation was used to obtain PBMC. The whole blood or buffy coat was mixed with 0.02 mM ethylenediaminetetraacetic acid (EDTA) (Sigma-Aldrich) and phosphate buffered saline (PBS) (Sigma-Aldrich, Saint Louis, MO, USA). After that, the mixture was layered on Histopaque-1077 (Sigma-Aldrich). Next, the cells were centrifuged at 400 g for 30 min at room temperature. The centrifuged PBMCs were transferred to clean tubes and washed three times in 1 × PBS (Sigma-Aldrich). The Bürker chamber was used to count the number of obtained PBMCs.

#### Sulforhodamine B Assay

Sulforhodamine B assay was used to determine the IC50 values of six commonly used first- (haloperidol) and second-generation (quetiapine, aripiprazole, risperidone, olanzapine and clozapine) antipsychotics (28). The exponentially growing PBMCs an initial density of 5 × 103/well were seeded into 96-well plates in triplicate. The drugs were added after 24 h with various concentrations. The mixture was incubated for 72 h at 37°C. Next, the PBMCs were fixed (Carnoy's solution; 30 min), twice washed in 1 × PBS, dried at room temperature, and finally stained with sulforhodamine B (SRB) (Sigma-Aldrich). The optical density was measured spectrophotometrically at 570 nm on an Infinite F50 microplate reader (Tecan, Männedorf, Switzerland). The IC50 values were calculated with GraphPad Prism 8.2 software (GraphPad Software, La Jolla, CA, USA), using four-parameter non-linear logistic regression. The doses corresponding to half IC50 and one-quarter IC50 were then determined.

#### Cell Culture

The PBMCs (at a density of 106 cells/mL) were suspended in RPMI-1640 enriched with antibiotics [1% penicillin/streptomycin (Sigma-Aldrich)], 2 mM L-glutamate (Gibco, Waltham, MA, USA), and 10% heat-inactivated fetal bovine serum (FBS) (Gibco). Next, suspended PBMCs were seeded in 48-well sterile (non-pyrogenic) flat bottom plates (Corning, Tewksbury, MA, USA). After that, the PBMCs were cultured in a 5% CO_2_ humidified atmosphere for 72 h at 37°C with quetiapine (1.8, 0.9, and 0.45 μg/mL); haloperidol (4.5, 2.25, and 1.12 μg/mL); aripiprazole (2.9, 1.45, and 0.725 μg/mL); risperidone (1.7, 0.85, and 0.425 μg/mL); olanzapine (1, 0.5, and 0.25 μg/mL); clozapine (2.8, 1.4, and 0.7 μg/mL) (all purchased from Tocris Bioscience, Bristol, UK). After 72 h of cells culture all supernatant samples were collected followed by freezing in aliquots at −80°C.

#### Quantitive RT-PCR (qRT-PCR)

The concentration (*A*260) and the purity (*A*260/*A*280) of isolated from 5 × 10^6^ PBMCs RNA by TRI Reagent® (Sigma-Aldrich) was measured at NanoDrop 2000 spectrophotometer (Thermo Scientific, USA). Next, RNA samples were immediately frozen at −80°C. Before analysis, cDNA was synthesized from the RNA samples (High-Capacity cDNA Reverse Transcription Kit; Applied Biosystems, USA), according to the manufacturer's protocol. Applied primers were previously designed in Primer3 Software, and then their sequences were confirmed by BLAST (Table 1). qRT-PCR was performed in duplicates, using Eco Real-Time PCR System (Illumina, USA). The reaction mixture contained: cDNA template, 0.2 nM of forward and reverse primers, and TaqMan® Fast Universal PCR Master Mix (Applied Biosystems, USA) in 10 μL of the final volume. The reactions were performed at 96°C for 2 min, followed by 40 cycles of 96°C for 5 s and 60°C for 30 s. The expression of receptor mRNAs was normalized to *ACTB* as the housekeeping gene.

**Table 1:** Sequences of the primers used in this study.

**Gene**	**Forward primer**	**Reverse primer**
D1R	AGCCATCATGAGCCACGAGGCTCC	TCTCCAGAGAGACGTCAGTGTCATAGTCCA
D2R	CCATGCTGTACAATACGCGCTAC	GATAACGGTGCAGAGTTTCATGTC
D3R	TGGTAAACTCCTCGGTCTCCAGA	CAGAGATGCCATAGCCCAGAGG
D4R	CCACCAACTCCTTCATCGTGAGCC	GATGGAGGCGGTGCACAGCA
5-HT 1AR	AGGCTGGTCCTACCCCTTGT	CGGCGTTGCGCTCATT
5-HT 2AR	AGCTGATATGCTGCTGGGTTTC	CCACCGGTACCCATACAGGAT
5-HT 3AR	GCCAACTGACGCCATCCT	ACCGCATTCCTCAGGTTCAC
ACTB	CTGGGACGACATGGAGAAAA	AAGGAAGGCTGGAAGAGTGC

#### Western Blotting

According to the manufacturer's protocol, the Trizol-isolated protein fraction concentration was quantified with the BCA Protein Assay Kit. Then lysates (30 μg) were separated on 10% SDS-PAGE gels (Bio-Rad, California, CA, USA) and blotted (120 min; 200 mA; 4°C) onto nitrocellulose membranes (Bio-Rad). To avoid non-specific antibody interactions, the membranes were blocked (5% BSA in PBS) for 2 h at RT. Next, the primary antibodies were added and incubated with the membrane overnight at 4°C. Finally, the membrane was washing incubated with horseradish peroxidase-conjugated secondary antibodies for 1 h at RT. Protein was detected with Pierce ECL Western Blotting Substrate on Kodak BioMax Light Film (Eastman Kodak, Rochester, NY, USA). The membrane was scanned (HP Scanjet G4050) and analyzed by ImageJ Software (USA) for protein level quantification. Follower primary antibodies: rabbit anti-D1 Abcam; No. ab20066, rabbit anti-D2 Abcam, No. ab85367, rabbit anti-D3 Abcam, No. ab155098, rabbit anti-D4 Abcam, No ab20424, rabbit anti-HT1AR Abcam, No.ab85615, rabbit anti-HT2AR Abcam, No.ab216959, rabbit anti-HT3AR Abcam, Noab13897, goat anti-GAPDH- Santa Cruz Biotech, CA, USA; No. I-19 and secondary antibodies (anti-goat and anti-rabbit antibodies conjugated with horseradish peroxidase) were purchased from Santa Cruz Biotech (Santa Cruz, CA, USA) were applied.

### Anthropometry and Body Composition

Basic anthropometric parameters were measured using standard procedures: height with a wall-mounted height measure to the nearest 0.5 cm, weight with a spring balance to the nearest 0.5 kg. Body mass index (BMI) was calculated as body weight [kg]/height [m]^2^. Waist and hip circumference were measured using a non-stretchable fiber measuring tape. Waist-to-hip ratio (WHR) was calculated as waist circumference divided by hip circumference.

Body composition was measured by a trained operator using two methods: bioimpedance analysis (BIA) and dual-energy X-ray absorptiometry (DXA), according to protocol adopted in our previous studies (29). The BIA was performed using a Maltron BIOSCAN 920-2-S Body Fat Analyzer (Maltron, Stafford, UK) multi-frequency (5, 50, 100, and 200 kHz) bioelectrical impedance analyzer. The DXA used a Lunar iDXA scanner (GE Healthcare, Chicago, IL, USA) with CoreScan software, version 15. DXA was used to measure TBF (total body fat), LBM (lean body mass), VAT (visceral adipose tissue) mass and VAT volume. TBF and LBM are expressed both in kilograms and as a percentage of total body mass. Fat mass index (FMI) was calculated as total body fat in kilograms (measured by DXA) divided by height in meters squared (kg/m^2^). The SAT (subcutaneous adipose tissue) and VAT cross-section areas (cm^2^) at the umbilicus level are measured as impedance and phase angle using the BIA analyzer and converted into the area using Maltron software.

### Statistical Analysis

Statistical procedures were performed with STATA 15.1 (StataCorp, Lakeway, TX, USA) and GraphPad Prism 8.2 (GraphPad Software, La Jolla, CA, USA). The level of significance was set at *p* < 0.05 (two-sided). For all continuous variables simple descriptive statistics [means, standard deviations, 95% confidence interval [95% CI]] were generated, while for discrete variables, the number of patients and percentages are given.

For all quantitative variables the normality of distribution was tested with the Shapiro-Wilk test. As the mRNA expression data was found to be highly skewed for all receptors, it was adjusted to a normal distribution by square root transformation before further analysis; this method of transformation was chosen empirically for best normality. This approach resulted in a normal distribution of all transformed variables with the exception of D_2_ and 5-HT3A receptor expressions. Therefore, transformed data were analyzed with Student's *t*-test, ANOVA and Pearson's correlation, while non-transformed data for D2 and 5-HT3A receptors was analyzed using non-parametric Wilcoxon rank-sum, Kruskal-Wallis, and Spearman's rank-order correlation tests. Linear regression models adjusted for smoking and analysis of covariance (ANCOVA) were used to examine the effect of clinical and anthropometric parameters and body composition. Linear regression models were run as backward step-wise models, i.e., they began with all variables included, and then the least useful predictor was iteratively removed, one-at-a-time, in order to get the best model with the full least squares model. In the regression models, receptor expression was used as the dependent variable, while the clinical variables (PANSS/CDSS scores) and smoking were the independent variables. Receptor expression was compared with clinical variables (dominance of positive or negative symptoms treatment with antipsychotics/antidepressants/mood stabilizers) using the aforementioned inter-group tests for means or medians, with the clinical variables used as dichotomous grouping variables.

A *post hoc* power analysis was performed for the most statistically significant results using G^*^Power 3.1 software (Heinrich-Heine-Universität, Germany) with an alpha error probability set at 0.05. This analysis revealed a sufficient power for the given sample size (1-β > 0.9 for all analyzed comparisons).

## Results

### Baseline Characteristics

Both study groups were highly comparable for demographic, clinical, anthropometric, and metabolic parameters (see Table 2 for details).

**Table 2:** Demographic and cardio-metabolic parameters of the study subjects.

	**Schizophrenia** ** (*n =* 27)**	**Control** ** (*n* = 29)**	***P***
Male	18 (66.7%)	20 (69.0%)	NS
Age [years]	38.6 ± 9.3	37.9 ± 10.6	NS
Smoking	19 (70.4%)	4 (13.8%)	<0.001
Smoking [pack-years]	10.9 ± 10.8	2.7 ± 6.7	<0.001
**COMORBIDITIES**			
Hypertension	5 (18.5%)	7 (24.1%)	NS
Diabetes	2 (7.4%)	0	NS
Dyslipidemia	8 (29.6%)	6 (20.7%)	NS
**ANTHROPOMETRIC PARAMETERS**			
Weight [kg]	79.8 ± 14.4	82.6 ± 18.4	NS
Body mass index [kg/m^2^]	27.3 ± 4.0	26.4 ± 5.4	NS
Waist circumference [cm]	99.0 ± 10.6	91.2 ± 16.1	0.038
Hip circumference [cm]	102.9 ± 8.0	102.3 ± 10.3	NS
Waist to hip ratio	0.96 ± 0.07	0.88 ± 0.08	<0.001
Systolic blood pressure [mm Hg]	122.4 ± 14.5	132.5 ± 17.9	0.02
Diastolic blood pressure [mm Hg]	78.9 ± 11.0	83.7 ± 11.1	NS
**LABORATORY TESTS**			
C-reactive protein [mg/dL]	2.5 ± 3.2	4.1 ± 8.5	NS
Glucose [mg/dL]	88.6 ± 19.6	88.23 ± 15.2	NS
Triglycerides [mg/dL]	133.9 ± 53.3	187.2 ± 129.2	NS
Total cholesterol [mg/dL]	197.2 ± 33.4	209.8 ± 44.1	NS
HDL cholesterol [mg/dL]	53.3 ± 13.9	52.1 ± 12.8	NS
LDL cholesterol [mg/dL]	114.5 ± 32.8	120.5 ± 36.1	NS
**BODY COMPOSITION**			
SAT area [cm^2^]	169.7 ± 75.2	150.5 ± 70.1	NS
VAT area [cm^2^]	142.5 ± 69.4	110.1 ± 69.5	NS
VAT mass [g]	1309.1 ± 713.4	1087.6 ± 1025.3	NS
VAT mass [% of body mass]	1.57 ± 0.72	1.16 ± 0.94	NS
VAT volume [cm^3^]	1387.7 ± 756.3	1152.8 ± 1086.9	NS
TBF [g]	27146.3 ± 8371.1	25385.3 ± 12278.4	NS
TBF [% of body mass]	33.5 ± 6.7	30.1 ± 9.2	0.042
LBM [g]	51684.6 ± 9480.6	52966.5 ± 10210.7	NS
LBM [% of body mass]	64.9 ± 8.6	66.1 ± 8.8	NS
Fat mass index [kg/m^2^]	9.3 ± 2.9	8.2 ± 4.2	NS

*Data given as: n (%) or mean ± standard deviation*.

*SAT, subcutaneous adipose tissue; VAT, visceral adipose tissue; TBF, total body fat; LBM, lean body mass*.

### Clinical Data

All schizophrenia patients were chronically ill, with an average duration of treatment of 16.1 ± 10.8 years and a mean number of hospitalizations of 10.3 ± 11.9. The mean number of acute psychotic episodes was 6.1 ± 4.2, while the meantime from the last hospitalization was 4.0 ± 7.1 months. Antipsychotic treatment in the schizophrenia group was heterogeneous (81.5% patients were on antipsychotic polypharmacy) and included second-generation antipsychotics (27 patients, 100%; most commonly olanzapine, clozapine, and aripiprazole) and first-generation antipsychotics (nine patients, 33.3%). Antipsychotics were administered in standard or above-standard doses, with the mean sum of DDD (defined daily dose) for individual antipsychotics equaling 2.8 ± 1.8. Six patients (22.2%) were also taking antidepressants (mostly serotonin-uptake selective serotonin reuptake inhibitors), while 13 individuals (48.4%) were taking mood stabilizers (mostly lamotrigine and valproate).

The severity of schizophrenia symptoms was low to moderate (total PANSS score 65.4 ± 15.3, P sub-score 15.3 ± 5.1, N sub-score 17.9 ± 4.5, G sub-score 32.1 ± 7.3), with 70.4% of patients having dominating negative symptoms. The mean severity of depressive symptoms (1.9 ± 1.9) was below the cutoff point for depression and low severity of depressive symptoms (CDSS score 1.9 ± 1.9) and no patients were classified as being depressed.

### Expression of Transcripts for Dopamine and Serotonin Receptors in PBMCs of Study Subjects

**Figure 1:**
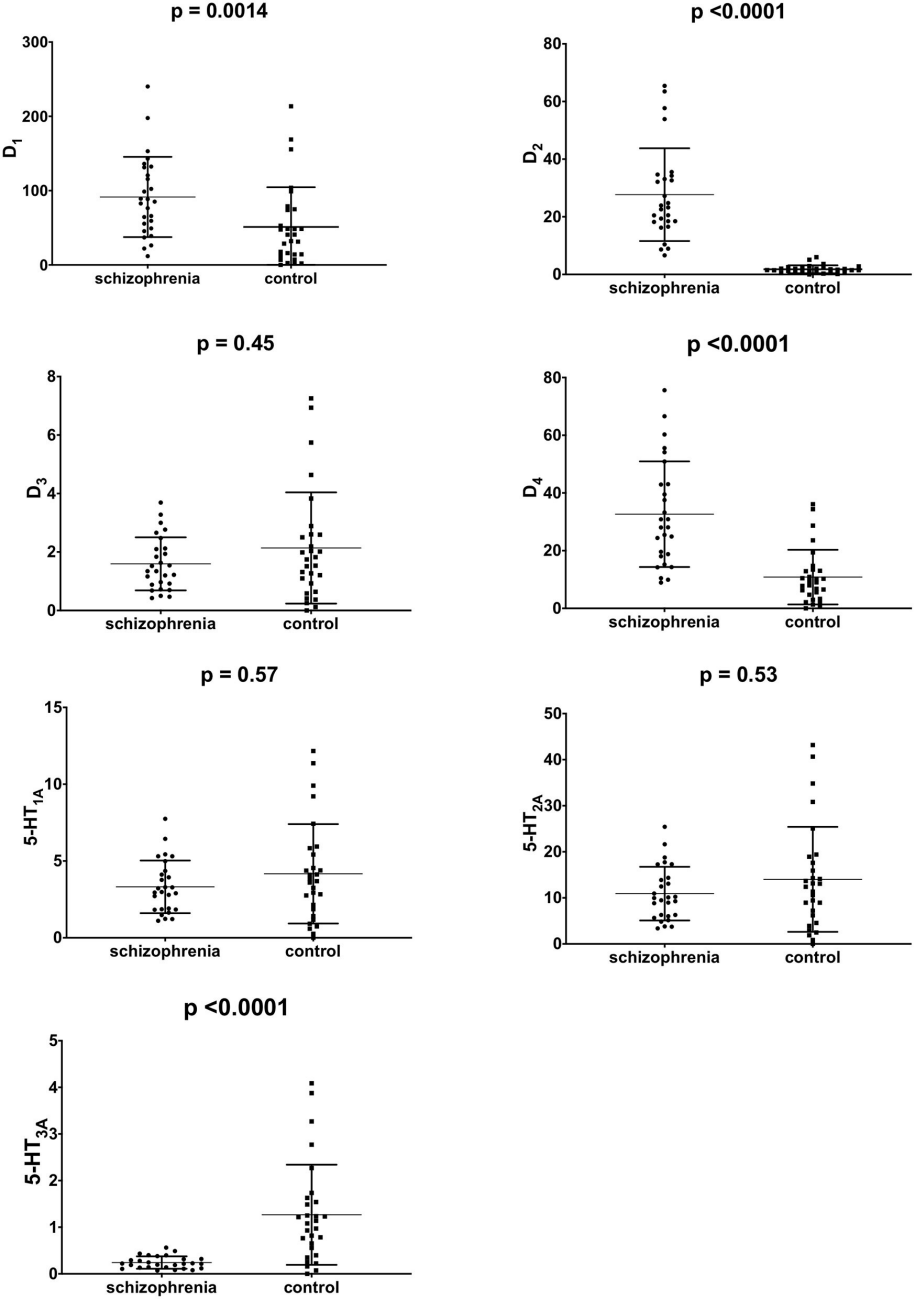
mRNA expression of dopamine D_1−4_ and serotonin 5-HT_1A−3A_ in PBMCs of study subjects (unadjusted for smoking). Middle horizontal line indicate mean, outer horizontal bars represent standard deviation.

The mRNA expression of dopamine D_1_, D_2_, D_3_, D_4_, and serotonin 5-HT_1A_, 5-HT_2A_, 5-HT_3A_ receptors in PBMCs were measured in all study subjects (Table 3). Significant inter-group differences were found; the schizophrenia group demonstrated higher D_1_, D_2_, and D_4_ receptor expression and lower 5-HT_3A_ receptor expression (Figure 1). Several strong correlations between the gene expression of individual receptors (Table 4). Interestingly, while correlations between D_1_ or D_2_ and other receptors were observed in the control group, no such correlations were present in the schizophrenia group.

**Table 3:** mRNA expression of dopamine D_1−4_ and serotonin 5-HT_1A−3A_ receptors in PBMCs of study subjects (unadjusted for smoking).

	**Schizophrenia** ** (*n* = 27)**	**Control** ** (*n* = 29)**	***P***
**D**_**1**_			
	91.46 ± 54.00 [70.10–112.82]	51.17 ± 53.42 [30.84–71.49]	0.001
**D**_**2**_			
	27.68 ± 16.09 [21.31–34.05]	1.76 ± 1.37 [1.24–2.28]	<0.0001
**D**_**3**_			
	1.60 ± 0.91 [1.23–1.96]	2.14 ± 1.90 [1.46–2.86]	0.45
**D**_**4**_			
	32.64 ± 18.31 [25.40–39.89]	10.84 ± 9.47 [7.24–14.44]	<0.0001
**5–HT**_**1A**_			
	3.32 ± 1.71 [2.64–4.00]	4.16 ± 3.23 [2.93–5.39]	0.57
**5–HT**_**2A**_			
	10.92 ± 5.82 [8.61–13.22]	14.01 ± 11.39 [9.68–18.34]	0.53
**5-HT**_**3A**_			
	0.24 ± 0.13 [0.19–0.29]	1.27 ± 1.07 [0.86–1.68]	<0.0001

*Data given as mean ± standard deviation [95% confidence interval]*.

**Table 4:** Correlations between mRNA expression of dopamine D_1−4_ and serotonin 5-HT_1A−3A_ receptors in PBMCs of study subjects (unadjusted for smoking).

**TOTAL STUDY GROUP**						
	**D_**1**_**	**D_**2**_**	**D_**3**_**	**D_**4**_**	**5-HT_**1A**_**	**5-HT_**2A**_**
D_2_	0.42^**^					
D_3_						
D_4_	0.33^*^	0.54^***^	0.51^***^			
5-HT_1A_	0.27^*^		0.98^***^	0.53^***^		
5-HT_2A_			0.99^***^	0.52^***^	0.99^***^	
5-HT_3A_		−0.38^**^	0.88^***^		0.85^***^	0.86^***^
**SCHIZOPHRENIA**						
	**D**_**1**_	**D**_**2**_	**D**_**3**_	**D**_**4**_	**5-HT**_**1A**_	**5-HT**_**2A**_
D_2_						
D_3_						
D_4_			0.99^***^			
5-HT_1A_			0.98^***^	0.98^***^		
5-HT_2A_			0.99^***^	0.99^***^	0.99^***^	
5-HT_3A_			0.99^***^	0.99^***^	0.99^***^	0.99^***^
**CONTROL GROUP**						
	D_1_	D_2_	D_3_	D_4_	5-HT_1A_	5-HT_2A_
D_2_	0.40^*^					
D_3_	0.51^**^	0.51^**^				
D_4_	0.52^**^	0.52^***^	0.99^***^			
5-HT_1A_	0.55^**^	0.61^***^	0.98^***^	0.99^***^		
5-HT_2A_	0.54^**^	0.58^***^	0.99^***^	0.99^***^	0.99^***^	
5-HT_3A_	0.53^**^	0.54^***^	0.99^***^	0.99^***^	0.99^***^	0.99^***^

*Data given as Pearson's correlation coefficient values*.

*** *p < 0.001*;

** *p < 0.01*;

* *p < 0.05*.

**Figure 2:**
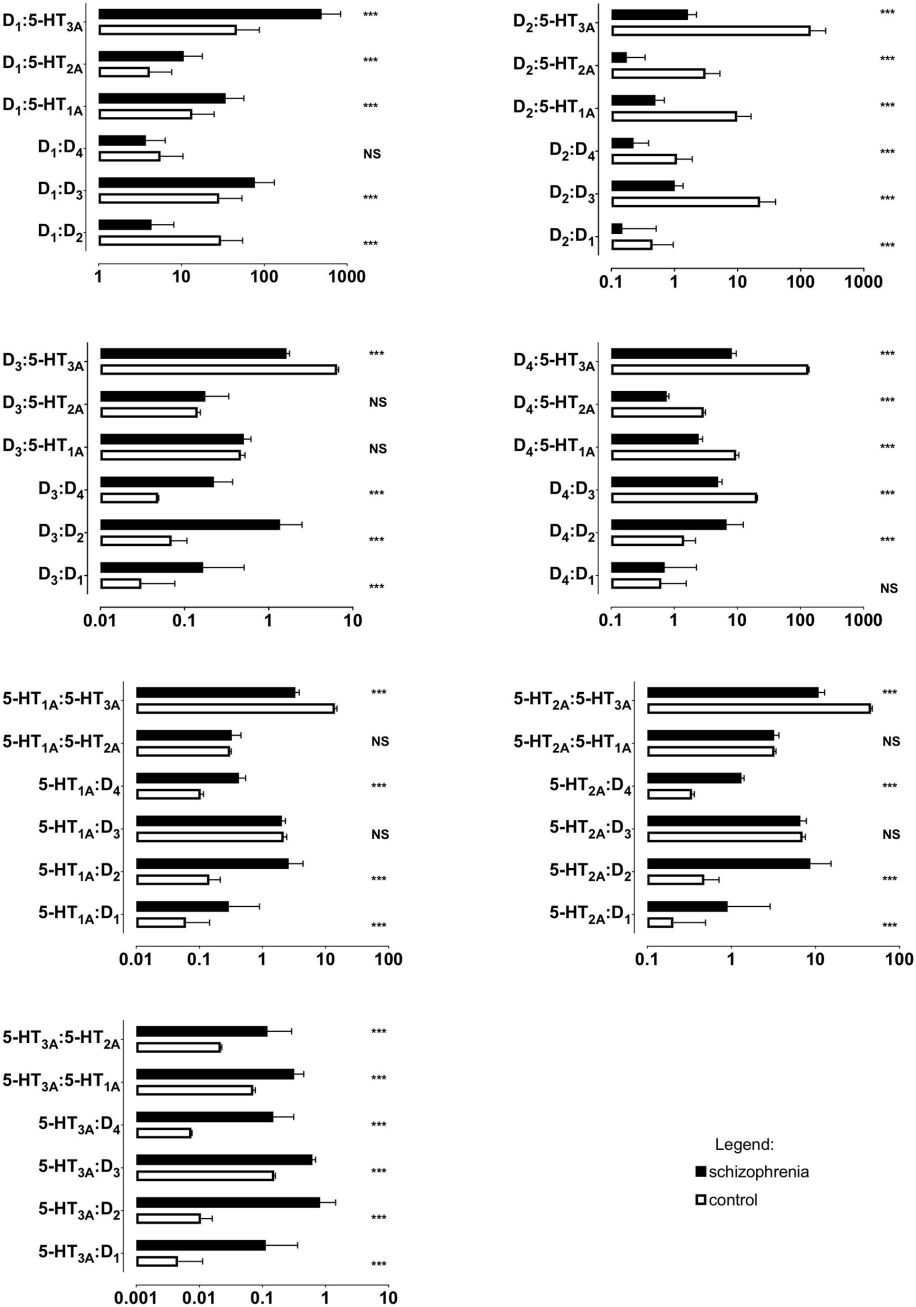
Ratios of mRNA expression of dopamine D_1−4_ and serotonin 5-HT_1A−3A_ receptors in PBMCs of study subjects (unadjusted for smoking). Horizontal bar represent mean and standard deviation. *** *p* < 0.001; NS, non-significant; X axes are log_10_ scaled.

All ratios of gene expression between individual receptors (D_1_:D_2_, D_1_:D_3_, D_1_:D_4_, etc.) differed significantly between the schizophrenia and control groups, except for D_1_:D_4_, D_3_:5-HT_1A_, D_3_:5-HT_2A_ and 5-HT_1A_:5-HT_2A_ (Table 5 and Figure 2); *p* < 0.001 for all comparisons.

**Table 5:** Ratios of mRNA expression of dopamine D_1−4_ and serotonin 5-HT_1A−3A_ receptors in PBMCs of study subjects (unadjusted for smoking).

	**D_**1**_**	**D_**2**_**	**D_**3**_**	**D_**4**_**	**5-HT_**1A**_**	**5-HT_**2A**_**	**5-HT_**3A**_**
D_1_		***	***	NS	***	***	***
SHZ		0.4 ± 0.5	0.1 ± 0.1	0.6 ± 0.9	0.1 ± 0.1	0.2 ± 0.3	0.01 ± 0.01
CON		0.1 ± 0.4	0.2 ± 0.3	0.7 ± 1.5	0.3 ± 0.6	0.9 ± 2.0	0.1 ± 0.2
D_2_	***		***	***	***	***	***
SHZ	4.4 ± 3.7		0.1 ± 0.1	1.4 ± 0.7	0.1 ± 0.1	0.5 ± 0.2	0.01 ± 0.01
CON	30.0 ± 24.7		1.4 ± 1.1	6.8 ± 5.7	2.6 ± 1.8	8.7 ± 6.6	0.8 ± 0.6
D_3_	***	***		***	NS	NS	***
SHZ	77.1 ± 55.2	22.7 ± 17.2		20.6 ± 0.3	2.2 ± 0.3	7.0 ± 0.5	0.2 ± 0.01
CON	28.6 ± 25.2	1.0 ± 0.3		4.9 ± 0.8	2.0 ± 0.3	6.6 ± 1.2	0.6 ± 0.1
D_4_	NS	***	***		***	***	***
SHZ	3.7 ± 2.6	1.1 ± 0.8	0.1 ± 0.1		0.1 ± 0.1	0.3 ± 0.1	0.01 ± 0.01
CON	5.6 ± 4.8	0.2 ± 0.2	0.2 ± 0.1		0.4 ± 0.1	1.3 ± 0.1	0.2 ± 0.2
5-HT_1A_	***	***	NS	***		NS	***
SHZ	34.2 ± 22.5	9.9 ± 6.5	0.5 ± 0.1	9.6 ± 0.9		3.2 ± 0.1	0.07 ± 0.01
CON	13.5 ± 11.3	0.5 ± 0.2	0.5 ± 0.1	2.4 ± 0.4		3.2 ± 0.4	0.3 ± 0.1
5-HT_2A_	***	***	NS	***	NS		***
SHZ	10.7 ± 7.2	3.1 ± 2.1	0.1 ± 0.1	2.9 ± 0.2	0.3 ± 0.1		0.02 ± 0.01
CON	4.2 ± 3.5	0.2 ± 0.2	0.2 ± 0.2	0.8 ± 0.1	0.3 ± 0.1		0.1 ± 0.2
5-HT_3A_	***	***	***	133.8 ± 3.4	***	***	
SHZ	493.4 ± 342.2	144.4 ± 104.4	6.5 ± 0.3		14.1 ± 1.1	45.6 ± 1.6	
CON	46.8 ± 40.5	1.6 ± 0.6	1.6 ± 0.1	8.2 ± 1.4	3.3 ± 0.5	10.9 ± 1.9	

*SHZ, schizophrenia group; CON, control group*.

*Data given as: mean ± standard deviation*.

*NS, non-significant*.

*** *p < 0.001*.

### Associations With Clinical Features of Schizophrenia

In the schizophrenia patients, pharmacological interventions were heterogeneous. The sub-groups of patients on individual medications being too small for accurate analysis of interactions between individual antipsychotics, antidepressants and mood stabilizers. The mRNA expression of the tested receptors did not differ between patients co-treated with antidepressants or mood stabilizers: both were tested using the t-test or the Wilcoxon rank-sum test, as well as linear regressions with treatment with antipsychotics, antidepressants and mood stabilizers being dichotomous variables. Similarly, no significant differences were observed with a subgroup of patients treated with multi-receptor antipsychotics (clozapine, olanzapine, quetiapine). The subgroup of patients treated with clozapine (*n* = 12, but only two on clozapine monotherapy) demonstrated significantly higher expression of D_2_ receptor mRNA (*p* = 0.041) compared with schizophrenia patients not taking clozapine. As this subgroup was very small, the result was confirmed using linear regression, with clozapine usage as the dichotomous variable (coefficient = 14.02, adjusted *R*^2^ = 0.16, *p* = 0.02). No such differences were observed for other receptors. Linear regression models adjusted for smoking found receptor expression to be associated only with the severity of depressive symptoms and not with general schizophrenia psychopathology (Table 6). No differences were observed for co-treatment with antidepressants, mood stabilizers or use of first-generation antipsychotics, or for treatment with multi-receptor antipsychotics (clozapine/olanzapine/quetiapine), treatment with clozapine or for dominance of positive or negative symptoms of schizophrenia (*p* > 0.05 in all cases).

**Table 6:** Linear regression models (adjusted for smoking) for the associations between severity of schizophrenia/depressive symptoms and mRNA expression or ratios of mRNA expression for dopamine and serotonin receptors in PBMCs of study subjects.

	**PANSS total**	**PANSS!!break P**	**PANSS N**	**PANSS G**	**CDSS**	***R*^2^**
D_3_	NS	NS	NS	NS	−0.27	0.31
D_4_	NS	NS	NS	NS	−5.54	0.32
5–HT_1A_	NS	NS	NS	NS	−0.51	0.31
5–HT_2A_	NS	NS	NS	NS	−1.74	0.31
5-HT_3A_	NS	NS	NS	NS	−0.04	0.31
D_4_:D_3_	NS	NS	NS	NS	0.09	0.25
D_4_:5-HT_3A_	NS	NS	NS	NS	−0.99	0.23
5-HT_1A_:D_3_	NS	NS	NS	NS	−0.08	0.26
5-HT_1A_:D_4_	NS	NS	NS	NS	0.01	0.26
5-HT_1A_:5-HT_2A_	NS	NS	NS	NS	0.01	0.25
5-HT_1A_:5-HT_3A_	NS	NS	NS	NS	0.33	0.26
5-HT_2A_:D_3_	NS	NS	NS	NS	0.16	0.26
5-HT_2A_:D_4_	NS	NS	NS	NS	0.01	0.25
5-HT_2A_:5-HT_3A_	NS	NS	NS	NS	0.48	0.25
5-HT_3A_:D_3_	NS	NS	NS	NS	0.01	0.25
5-HT_3A_:D_4_	NS	NS	NS	NS	0.01	0.25

*Data shown as β (standardized coefficient), R^2^ = coefficient of determination*.

*All p < 0.05*.

*NS, non-significant*.

### Potential Confounders

A series of ANCOVA tests were performed to identify any possible confounding effect of tested variables (i.e., sex, age, smoking status, laboratory tests, anthropometric parameters, and body composition). With the exception of smoking, all the above reported inter-group comparisons provided the same test results when the confounders were included as co-variates. As the levels of expression of D_1_, D_2_, D_3_, D_4_, and 5-HT_3A_ mRNAs were significantly higher in smokers (*p* < 0.05), inter-group comparisons were adjusted for the number of smoked cigarettes per day and duration of smoking. This adjusted comparison confirmed that the schizophrenia group demonstrated higher expression of D_2_ and D_4_ receptors (*p* < 0.001), but lower expression of D_3_, 5-HT_1A_, 5-HT_2A_, and 5-HT_3A_ receptors (*p* < 0.01). The initial difference in D_1_ expression was no longer significant after this adjustment. All analyses for dopamine and serotonin receptor expression ratios were also adjusted this way. As expected, smoking was the only significant confounder: the D_2_:D_1_, D_3_:D_1_, 5-HT_1A_:D_1_, 5-HT_2A_:D_1_, and 5-HT_3A_:D_1_ ratios were no longer significant after adjusting for smoking.

### The Effect of Antipsychotics on Dopamine and Serotonin Receptor Expression in PBMCs

**Figure 3:**
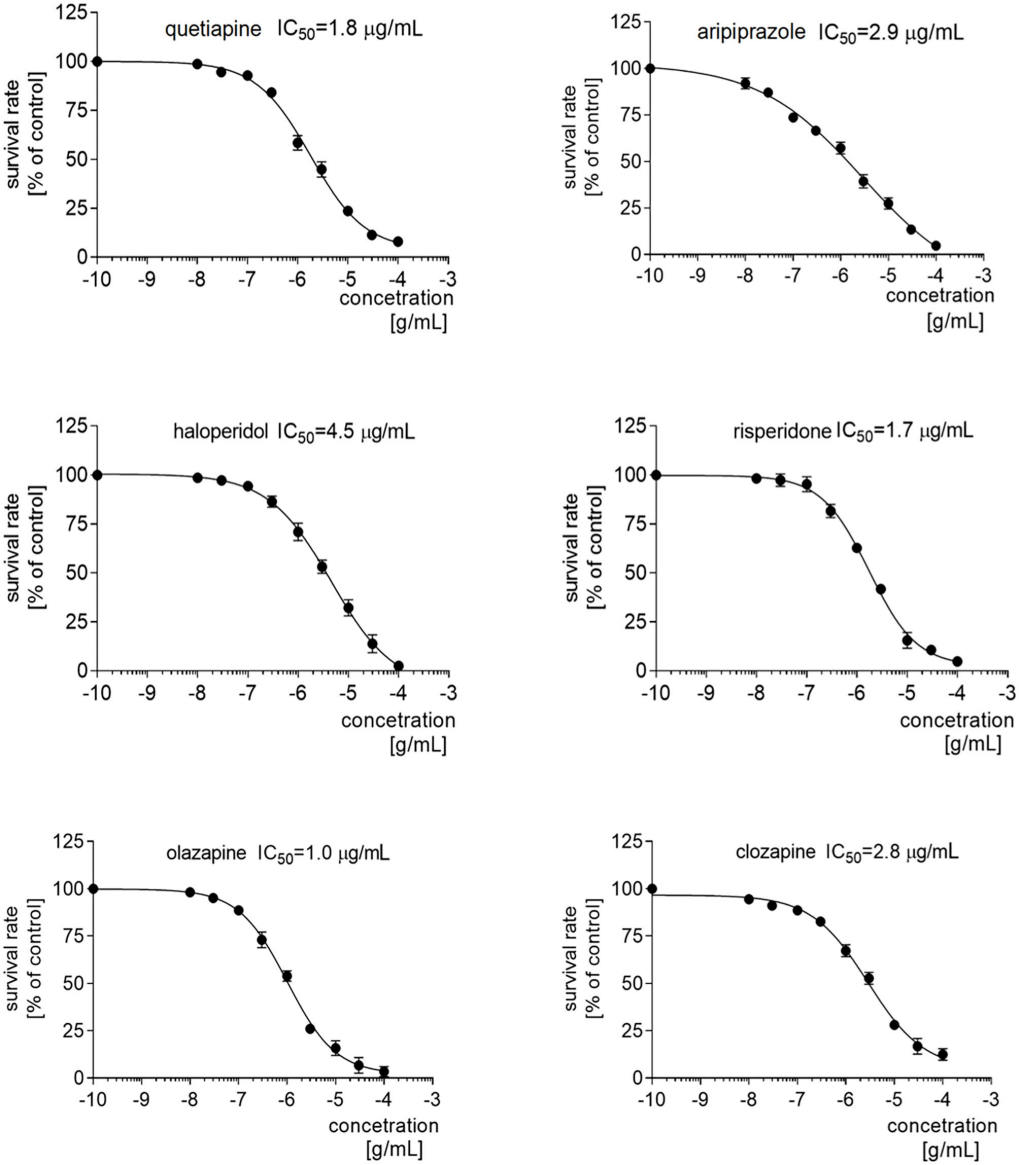
IC_50_ profiles of the PBMCs after treatment with antipsychotics. Graphs representing the proliferation of PMBCs treated with different concentrations of quetiapine, haloperidol, aripiprazole, risperidone, olanzapine, or clozapine. Above the graph, the IC50 value for each drug is presented.

The rationale for this experiment was briefly given in the Introduction section. Firstly, quetiapine, haloperidol, aripiprazole, risperidone, olanzapine, and clozapine were screened based on the sulforhodamine assay for their effects on the viability of the PBMCs, obtained from the healthy donors. Each of drugs decreased PMBC proliferation in a dose-dependent manner, with IC_50_ values of 1.8 μg/mL for quetiapine, 4.5 μg/mL for haloperidol, 2.9 μg/mL for aripiprazole, 1.7 μg/mL for risperidone, 1.0 μg/mL for olanzapine, and 2.8 μg/mL for clozapine (Figure 3). Therefore, in the following determination of dopamine and serotonin receptor expression via qRT-PCR and protein level via Western blot, the drugs were added at a concentration corresponding to IC_50_, half IC_50_, and one-quarter IC_50_.

**Figure 4:**
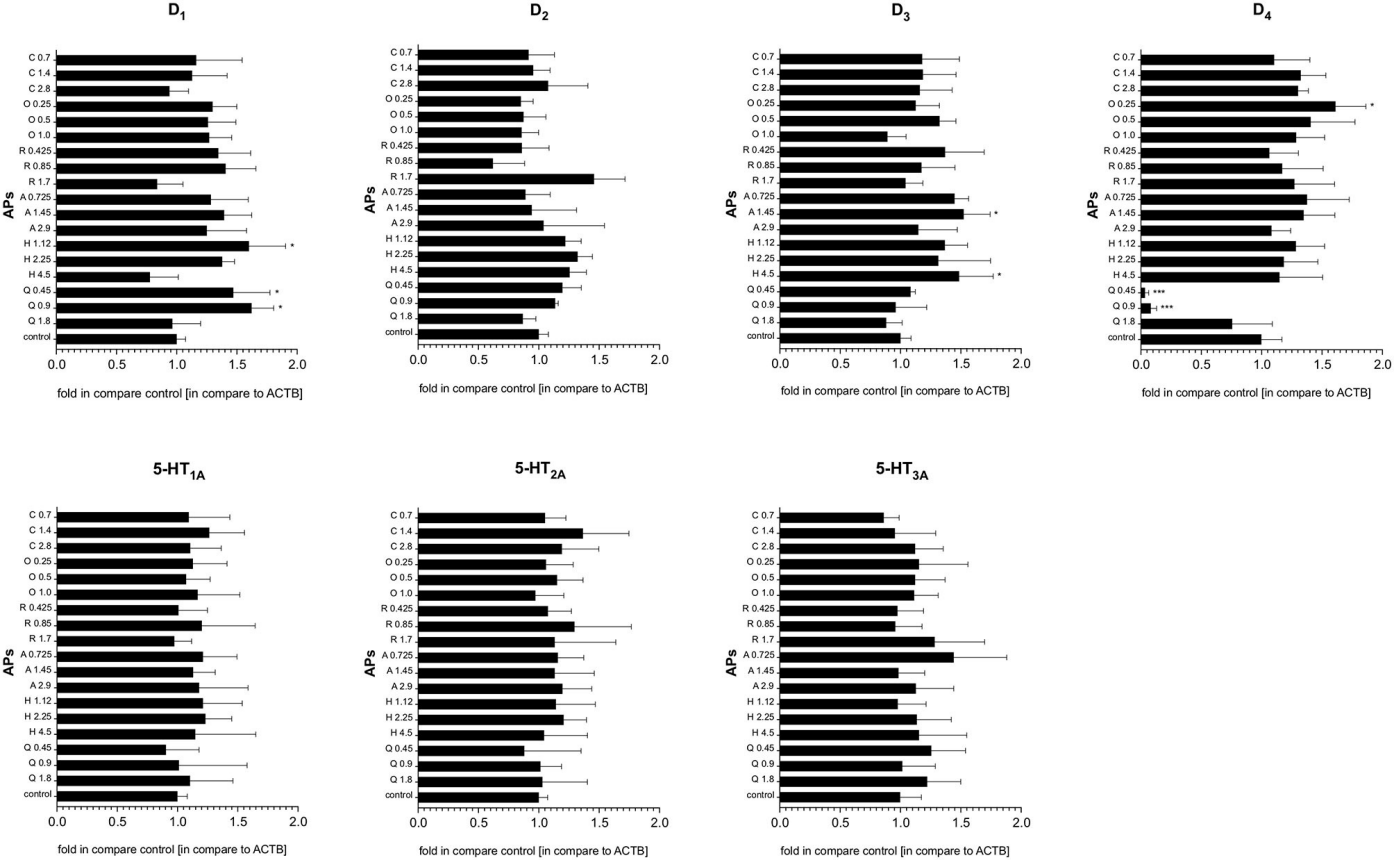
The effect of antipsychotic treatment on dopamine D_1−4_ and serotonin 5-HT_1A−3A_ receptors mRNA expression in PBMCs. Graphs represent the relative gene expression of dopamine (D_1−4_) and serotonin (5-HT_1A−3A_) receptors in PBMCs treated *in vitro* with antipsychotics (APs): quetiapine (Q), haloperidol (H), aripiprazole (A), risperidone (R), olanzapine (O), or clozapine (C) in dose corresponding to IC_50_, half of the IC_50_ and a quarter of IC_50_. Constitutive receptor expression served as the control. All results were normalized to ACTB. Bars represent mean and standard deviation. *p < 0.05; ***p < 0.001.

Generally, the qRT-PCR analysis revealed that treatment with quetiapine, risperidone, haloperidol, olanzapine, aripiprazole or clozapine did not induce any statistically significant modulation of serotonin 5-HT_1A_, 5-HT_2A_, and 5-HT_3A_ and dopamine D_2_ receptor gene expression in PBMCs. However, the D_1_ receptor was upregulated after quetiapine or haloperidol treatment, aripiprazole and haloperidol slightly enhanced the expression of the D_3_ receptor, while olanzapine upregulated D_4_ expression. Interestingly, only quetiapine treatment induced a significant decrease in dopamine D_4_ mRNA expression (Figure 4).

**Figure 5:**
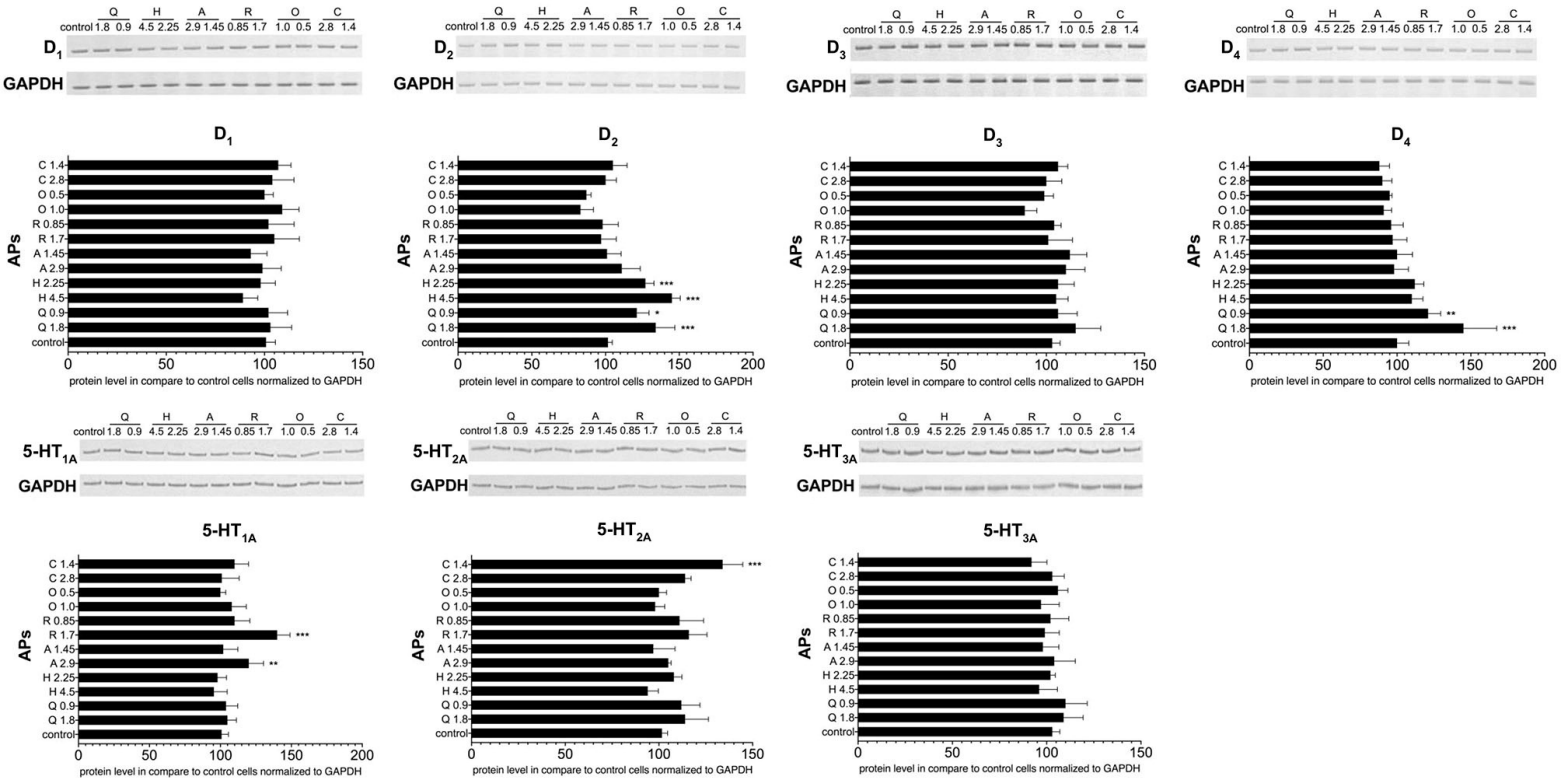
The effect of antipsychotic treatment on dopamine D_1−4_ and serotonin 5-HT_1A−3A_ receptors protein expression in PBMCs. Graphs show the relative protein level of dopamine (D_1−4_) and serotonin (5-HT_1A−3A_) receptors in PCMBs treated *in vitro* with antipsychotics (APs): quetiapine (Q), haloperidol (H), aripiprazole (A), risperidone (R), olanzapine (O), or clozapine (C) in doses corresponding to IC_50_ and one of a quarter of IC_50_. Constitutive receptor expression served as the control. All results were normalized to GAPDH (loading control). One representative blot of the three independent experiments is shown. Bars represent mean and standard deviation. *p < 0.05; **p < 0.01; ***p < 0.001.

The next stage examined the effect of antipsychotic treatment on dopamine and serotonin receptor expression in PBMCs on the protein level. Western blot analysis found higher concentrations of quetiapine and haloperidol to induce upregulation of the D_2_ receptor, and quetiapine to upregulate the D_4_ receptor. In turn, the protein level of the 5-HT_1A_ receptor was higher after aripiprazole or risperidone stimulation, whereas the protein level of the 5-HT_2A_ receptor was higher after 1.14 mg/mL clozapine (Figure 5).

## Discussion

This is the first study to examine the profile of mRNA expression of the dopamine D_1_, D_2_, D_3_, and D_4_ and serotonin 5-HT_1A_, 5-HT_2A_, and 5-HT_3A_ receptors in PBMCs in medicated patients with schizophrenia, and to perform an *in vitro* analysis of the effects of six major antipsychotics on the receptor expression in PBMCs.

Our results indicate that the mRNA expression of the inhibitory (D_2_ and D_4_) receptors differed significantly between medicated patients with schizophrenia and controls. Although the expression of the inhibitory D_1_ receptor was found to be higher, this difference was no longer significant after adjusting for smoking. Previous studies have reported the effects of smoking on the mRNA expression of dopamine receptors D_1_ and D_2_ in rat brains and D_3_ in human blood lymphocytes (30, 31). Our present study of three serotonin receptors found significantly lower expression of 5-HT_3A_ receptor mRNA in schizophrenia, and lower expression of 5-HT_1A_ and 5-HT_2A_ receptor mRNA after adjustment for smoking. Our findings also indicate that patients with schizophrenia demonstrate varying profiles of receptor expression ratios.

Previously, Zvara et al. found that D_2_ receptor mRNA is over-expressed in PBMCs in a small sample (*n* = 13) of drug-naive/drug-free schizophrenic patients, and suggest that this expression may serve as a potential diagnostic marker for schizophrenia (32). Similarly, our present observations on patients receiving antipsychotic treatment indicate that D_2_ receptor mRNA expression was significantly higher in schizophrenia patients (27.68 ± 16.09 vs. 1.76 ± 1.37, *p* < 0.001). The increased expression of D_2_ receptors in the brain may provoke hypersensitivity to dopamine, which is responsible for hallucinations (33) and the cognitive dysfunctions of schizophrenia, via persistent abnormalities in prefrontal cortex functioning (34).

Ahmadian et al. suggest that the peripheral expression of D_3_ receptors may also serve as a potential biomarker for schizophrenia, but only in patients not taking antipsychotics (35). They report increased expression of D_3_ receptors in schizophrenia, but only in a small (*n* = 15) subgroup of drug-free patients; in patients taking antipsychotics, D_3_ expression was comparable with the control group. Similar results have been demonstrated for the D_3_ and D_5_ receptors by Kwak et al. (36). Urhan-Kucuk et al. report higher D_3_ receptor levels in patients with prominent symptoms of disorganized behavior than in those with the paranoid subtype of schizophrenia, but not healthy controls (37). Finally, Vogel et al. report a reduced level of D_3_ mRNA in medicated patients with schizophrenia (38). No such difference was found between schizophrenia patients and healthy subjects for this receptor in the present study, which may be due to the fact that all study patients were already on antipsychotic treatment. Therefore, it remains inconclusive whether changes in D_3_ receptor expression could be used as a biomarker of potential response to this treatment.

Our results also reveal increased expression of D_4_ receptors in schizophrenia (32.64 ± 18.31 vs. 10.84 ± 9.47, *p* < 0.001). These receptors may be involved in the cognitive symptoms of schizophrenia, which are currently considered as the core symptoms of this condition (39, 40). The association is probably mediated by impaired GABAergic transmission (modulated via the D_4_ receptor), neuregulin/ErbB4 signaling pathways (also modulated by the D_4_ receptor), leading to dysregulation of the gamma frequency network oscillations and cognitive dysfunctions (41). Rocc et al. suggest that D_4_ receptor mRNA expression in PBMC may serve as a peripheral marker of the central dopaminergic function in major depression (42); they note that the expression of D_4_ receptors is reduced in untreated depression and returns to normal after treatment with paroxetine, but only when patients have achieved a significant improvement of depressive symptoms. Hence, the question of whether changes in D_4_ receptor expression could be used as a biomarker of potential response to antipsychotic treatment remains open.

Increased D_2_ receptor expression combined with unaltered D_1_ receptor expression may lead to an improper activation profile, i.e., high activation of D_2_ receptor and low activation of D_1_ receptor. Avery and Krichmar propose that such a profile may trigger noise in the neurons of the prefrontal cortex, which may result in working memory deficits and other symptoms of schizophrenia (43). In the present study, no correlations were found between D_1_ or D_2_ and other studied receptors compared with the control group (Table 4), while similar correlations were found for the D_3_, D_4_, 5-HT_1A_, 5-HT_2A_, and 5-HT_3A_ receptors. This may be explained by an imbalance between D1 and D2 receptor expression during schizophrenia.

Schizophrenia is characterized by a loss of serotonin 5-HT_2A_ receptor expression from the frontal cortex (44) and an increased density of 5-HT_1A_ receptors (12). This is only partly reflected by our results, which indicate only reduced expression of the 5-HT_2A_ and 5-HT_1A_ receptors. The lack of any increase in 5-HT_1A_ receptor expression could be at least partly explained by the heterogeneous composition of antipsychotic treatment in our study group, since antipsychotics differ in their effects on 5-HT_1A_ receptor expression (45). Studies suggest that 5-HT_3A_ receptors are not involved in in the pathogenesis of schizophrenia (46). Also, their expression may change during antipsychotic treatment, but only in certain subgroups of patients: Chen et al. report such changes only in responders to risperidone (47).

Before adjustments for smoking, our linear regression models showed numerous associations between the expression of individual receptors or the ratios of expression of these receptors and the severity of schizophrenia symptoms; most of these variables being associated with negative and total PANSS scores. However, when these models were adjusted, only associations between dopamine D_3_, D_4_ and serotonin 5-HT_1A−3A_ receptors and the severity of depressive symptoms remained significant. This may indicate that these receptors may also be involved in the pathogenesis of depressive symptoms. Previous studies have shown that D_3_ (48), D_4_ (49), and serotonin receptors (50) may all be associated with the pathophysiology of affective symptoms. In our study, the severity of depressive symptoms was found to be low; therefore, it was difficult to analyze the effect of depression, and further studies in schizophrenia patients with more prominent mood symptoms are needed to clarify this. Also, the impact of smoking is a significant finding considering that most patients with schizophrenia smoke.

Our series of *in vitro* experiments tested the effect of first- (haloperidol) and second-generation (quetiapine, aripiprazole, risperidone, olanzapine and clozapine) antipsychotics on the expression of dopamine D_1_, D_2_, D_3_, and D_4_ and serotonin 5-HT_1A_, 5-HT_2A_, and 5-HT_3A_ receptors in PBMCs. The IC_50_ values for each of the antipsychotics were identified previously. Our findings might be highly useful for future studies of cytotoxic activity of antipsychotics in PBMC models. Of the six studied antipsychotics, the lowest IC_50_ for PBMC growth was found for olanzapine (1.0 μg/mL) and highest for haloperidol (4.5 μg/mL), with aripiprazole and clozapine in between. Interestingly, while being pharmacologically similar to olanzapine, the IC_50_ value for clozapine was almost three times higher (2.8 μg/mL). The highest IC_50_ was found for haloperidol, which would suggest the lowest cytotoxic activity for PBMCs; this is particularly interesting in the light of the ongoing debate on potential neurotoxicity of antipsychotics. Current data suggest that first-generation antipsychotics are more likely to induce neurotoxicity, leading to extrapyramidal side effects (51), but second-generation antipsychotics may also trigger some neurodegenerative process (52).

Regarding the effect on receptors, some studies suggest that antipsychotics might affect gene expression in PBMCs and that differences exist between antipsychotic classes or types: for example, 5-HT_3A_ gene expression is significantly increased by olanzapine, but not haloperidol (21). However, Taraskina et al. did not identify significant changes in the expression of 5-HT_2A_ or D_4_ genes in PBMCs after 28 days of treatment with haloperidol or olanzapine (53). Also, successful treatment with risperidone has recently been found to increase the 5-HT3A receptor gene expression in patients with paranoid schizophrenia, indicating that the 5-HT3A receptor may be involved in the mechanism of risperidone effect (47). Our results are mixed in this regard. qRT-PCR analysis suggests that treatment with quetiapine, risperidone, haloperidol, olanzapine, aripiprazole, or clozapine does not induce any statistically significant modulation of the mRNA expression of all three tested serotonin receptors and the dopamine D_2_ receptor. The mRNA expression of other dopamine receptors was selectively reduced by quetiapine (D_4_), and enhanced by quetiapine and haloperidol (D_1_), aripiprazole and haloperidol (D_3_) or olanzapine (D_4_). At the protein level, the levels of D_2_ (quetiapine and haloperidol), D_4_ (quetiapine), 5-HT_1A_ (risperidone and aripiprazole), and 5-HT_2A_ (clozapine) receptor protein were increased, but only at high concentrations, i.e., up to the IC_50_ values. Thus, it is questionable whether clinically non-toxic doses of antipsychotics were able to induce such changes in our study subjects. This suggests that antipsychotics may only have a limited influence on the expression of dopamine or serotonin receptors in PBMCs, and that the observed profile of receptor mRNA expression may reflect the underlying pathophysiological process of schizophrenia.

There are several limitations of this study that have to be taken into consideration when interpreting its results. One of the most important limitations is that the sample size is relatively small; therefore, a larger replication study is required to confirm the results. Also, there is a potential issue of whether peripheral expression of dopamine and serotonin receptor mRNA reflects central expression, i.e., in the brain. The current opinion is that blood cells can be used *in vitro* for experimental studies of brain-expressed transcripts in both physiological and pathological states (54) and that peripheral expression reflects about 20% of the transcripts expressed in brain tissues. Another study indicates that for many genes, their peripheral gene expression may be a useful surrogate for gene expression in the brain (25). In addition, genes expressed in the brain have also been found to be expressed in PBMCs, and some show co-expression or similar expression in the same individuals, supporting their use as a surrogate tissue for gene expression profiling in schizophrenia (55). Specifically, for our study, such associations were found for D_2_ receptors in humans (25), D_3_ and 5-HT_2A_ receptors in rats (56).

A second limitation was that mRNA expression was only measured at one time point, so longitudinal changes and their associations with clinical features remain unknown. Moreover, all study patients were on antipsychotic pharmacotherapy, with some also receiving antidepressive and mood stabilizing medication. However, the fact that no antipsychotics were found to have any significant effects on PBMC dopamine and serotonin receptors, on neither the mRNA or protein level, suggests that the effect of treatment may be negligible and any observed differences may be disease specific. Consequently, further studies are needed on groups receiving more homogeneous treatments or with treatment-naïve patients, and more clinical features should be assessed (e.g., cognitive symptoms). It is also essential to test the schizophrenia specificity of these results, and hence replication studies with bipolar disorder and unipolar depression patients are needed. Also, although our *post hoc* analysis revealed sufficient power for the study sample size, a larger study sample would improve the power of these results. A minor limitation is also related to the low severity of the schizophrenia symptomatology (mean PANSS score 65), thus limiting chances for detecting the effect of clinical symptoms. Finally, since schizophrenia is a complex and heterogeneous disorder, it is probable that certain types of peripheral blood biomarkers may only define subsets of people with this disorder, and that these biomarkers could also potentially be used for dissecting schizophrenia into biologically-based subtypes.

## Conclusions

We propose that schizophrenia may be characterized by a certain pattern in the expression of dopamine and serotonin receptors at the mRNA level, and that a specific set of these receptors, rather than individual ones, might be useful as a potential diagnostic biomarker for schizophrenia. However, this pattern may demonstrate partial overlap with affective disorders, as the two conditions display similar profiles of individual dopamine or serotonin receptor expression, and hence clusters of receptors should be analyzed for increased specificity.

## Data Availability Statement

The original contributions presented in the study are included in the article/Supplementary Materials, further inquiries can be directed to the corresponding author/s.

## Ethics Statement

The studies involving human participants were reviewed and approved by Bioethics Commission of the Medical University of Lodz, Poland. The patients/participants provided their written informed consent to participate in this study.

## Author Contributions

AW, KS, and EB-B designed the study. ES, AŁ, EK, and JA collected data and performed laboratory experiments. AW analyzed data. AW, KS, EB-B, and EK wrote the main manuscript text. KS prepared Figures 4, 5. All authors reviewed the manuscript.

## Conflict of Interest

The authors declare that the research was conducted in the absence of any commercial or financial relationships that could be construed as a potential conflict of interest.
